# Aβ1-25-Derived Sphingolipid-Domain Tracer Peptide SBD Interacts with Membrane Ganglioside Clusters via a Coil-Helix-Coil Motif

**DOI:** 10.3390/ijms161125955

**Published:** 2015-11-03

**Authors:** Yaofeng Wang, Rachel Kraut, Yuguang Mu

**Affiliations:** School of Biological Sciences, Nanyang Technological University, 60 Nanyang Drive, 637551 Singapore, Singapore; ywang22@e.ntu.edu.sg (Y.W.); rskraut@ntu.edu.sg (R.K.)

**Keywords:** Sphingolipid binding domain, lipid rafts, molecular dynamics simulation

## Abstract

The Amyloid-β (Aβ)-derived, sphingolipid binding domain (SBD) peptide is a fluorescently tagged probe used to trace the diffusion behavior of sphingolipid-containing microdomains in cell membranes through binding to a constellation of glycosphingolipids, sphingomyelin, and cholesterol. However, the molecular details of the binding mechanism between SBD and plasma membrane domains remain unclear. Here, to investigate how the peptide recognizes the lipid surface at an atomically detailed level, SBD peptides in the environment of raft-like bilayers were examined in micro-seconds-long molecular dynamics simulations. We found that SBD adopted a coil-helix-coil structural motif, which binds to multiple GT1b gangliosides via salt bridges and CH–π interactions. Our simulation results demonstrate that the CH–π and electrostatic forces between SBD monomers and GT1b gangliosides clusters are the main driving forces in the binding process. The presence of the fluorescent dye and linker molecules do not change the binding mechanism of SBD probes with gangliosides, which involves the helix-turn-helix structural motif that was suggested to constitute a glycolipid binding domain common to some sphingolipid interacting proteins, including HIV gp120, prion, and Aβ.

## 1. Introduction

**Figure 1:**
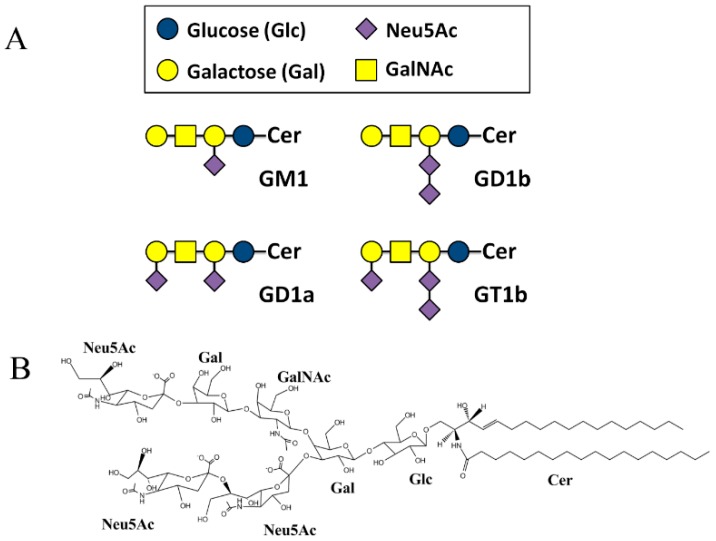
(**A**) Structure of the major brain gangliosides; (**B**) Detailed chemical structure of gangliosides GT1b. Cer, ceramide.

Sphingolipid-containing microdomains in cellular plasma membrane play important roles in signaling, endocytic, and secretory pathways [1,2]. Glycosphingolipids (GSLs) constitute a large and diverse class of lipids with carbohydrate-containing head groups. These complicated lipids are important mediators of pathogen infection [3,4], providing a platform for bacteria [5,6], virus [7], and toxins [8] to enter the host cells. The ganglioside family comprises glycosphingolipids having a common sphingolipid backbone with between one and three hexose sugars in the head group, to which variable numbers of sialic acids can be attached (Figure 1A). In human central nervous system (CNS), GM1, GD1a, GD1b and GT1b constitute 97% of all gangliosides [9]. GM1 with one sialic acid was the first to be identified and is the best characterized ganglioside so far (reviewed by Sandhoff *et al.* [10]). Recently, the trisialo-ganglioside GT1b (Figure 1B) was reported to have a different distribution from GM1 in the CNS [11]. GT1b is present in brain tumor metastasis [12], and is also the likely receptor for tetanus toxin [13], myelin-associated glycoprotein [14], and botulinum neurotoxin [15]. The interactions between GT1b and its ligands have been studied through both experimental and molecular modeling tools [16,17,18].

It has been proposed that toxin-membrane interactions are mediated by a discrete sphingolipid binding motif rich in aromatic amino acids containing one or more key basic residues [19,20]. This loosely-defined domain was suggested to exist in Aβ [21], HIV gp120 [22], prion [23], Shiga toxin [24], as well as more recently in α-synuclein [25]. Aβ peptide, which generally consists of a 40 or 42 amino acid cleavage product of the trans-membrane amyloid precursor protein (APP) protein [26], is thought to accumulate first into oligomers and then fibrils as a consequence of interactions with sphingolipids in raft micro-domains, in particular GM1, GT1b and probably other gangliosides [27,28,29,30,31,32,33]. There is abundant support for the idea that sialic acid containing glycosphingolipids (gangliosides), such as GM1, could affect the conformations of Aβ peptide [30,34,35]. Fantini and coworkers suggested that a helix-turn-helix region identified as the sphingolipid interacting domains in Aβ, HIV gp120, and prion all adopt a similar conformation during sphingolipid binding with GalCer and GM1 [19,23,36].

In a recent series of studies, a fluorescently-tagged variant of Aβ_1–25_, referred as to Sphingolipid Binding Domain (SBD) peptide, was constructed and characterized as a ganglioside and sphingolipid domain tracer in cellular and artificial membranes [37,38,39,40]. A mutation of K16E was also examined for sphingolipid binding [20]. Surprisingly, this Aβ_1–25_ variant showed a similar ganglioside preference as the Aβ_1–40_ peptide [28,41], even in the absence of the sequence from 26–40. Serendipitously, this provides the benefit of removing the β-sheet-prone segment of the peptide, which is thought to induce aggregation and produce cellular toxicity [31,32,42], while apparently retaining the sphingolipid binding function [38]. In these studies, the diffusion behaviors of SBD variants, monitored by fluorescence correlation spectroscopy (FCS), showed similar mobility characteristics to existing membrane raft markers that have also been characterized by FCS, such as CtxB [37,40].

On the other hand, the fluorescently tagged Aβ_1–25_ probe SBD showed a higher binding affinity to GT1b than GM1 at neutral pH, suggesting that the probe have different binding characteristics to GT1b and GM1 dependent on pH environments. Moreover, the cholesterol-dependent cell uptake and trafficking pathways of SBD were distinct from that of known raft markers [38,39]. These results have established fluorescently-labeled SBD probe as a tracer of domain behaviors for sphingolipid-containing microdomains in membrane, as well as in cell uptake and trafficking pathways. Cholesterol is reported to enhance the binding between Aβ peptide and GM1 by tuning the ganglioside’s conformation with hydrogen bonds [43] through CH–π interactions [44,45]. CH–π interactions have been proposed to play critical roles in maintaining biomolecular structures and involving in their biological functions [46]. They have been well studied in small-molecule systems [47,48] as well as protein molecules [49]. Compared to hydrogen bonds (NH:O=C, ~10 Kcal/mol), CH–π interactions are weak, for instance CH_4_–π is less than 0.25 Kal/mol.

The early binding process between full length Aβ and GM1 has also been investigated by MD simulations [50,51]. However, the molecular interactions between SBD and poly-sialic gangliosides, such as GT1b, remain unclear. Such interactions may mediate membrane domain-specific recognition and characteristic diffusion. Moreover, no structural information of fluorescently tagged Aβ peptide in the folding and binding with GT1b is available.

In this study we applied extensive molecular dynamics (MD) simulations to explore the detailed mechanism of the SBD–GT1b interaction with three questions in mind: (i) How do SBD monomers interact with the preferred ganglioside, GT1b, in the membrane? (ii) What are the key amino acids mediating the binding? (iii) Do fluorescent dye and linker molecules influence the gangliosides binding configurations of SBD probe? Such a mechanistic study is beneficial not only for the future design of novel sphingolipid and microdomain tracers, but also for understanding the recognizing mechanisms of pathogen invasion into cells.

## 2. Results

Previously, SBD peptide was suggested to bind to gangliosides in the presence of sphingomyelin and cholesterol using surface plasmon resonance and fluorescence correlation spectroscopic assays [37,38]. GT1b was found to have a marginally higher binding at neutral pH than other common brain gangliosides, such as GM1, GD1a, and GQ1b [20,38]. Our simulations employed the same 1-palmitoyl-2-oleoylphosphatidylcholine (POPC), sphingomyelin (18:0) (SM) and cholesterol (CHOL) composition used in these experimental studies. Plasma membrane-like POPC bilayer systems with four concentrations of GT1b (12%, 8%, 4% and 0%) were equilibrated to determine the best conditions for SBD binding. Key biophysical parameters calculated from our simulations, including the area per lipid, the bilayer thickness, and the lateral diffusion constants which characterize the GT1b containing raft-like POPC lipid bilayer systems, are close to those from the experimental and lipid raft model simulation studies (Table S1 and Figure S1).

Two types of SBD probes were employed in the simulations, including Aβ_1–25_ SBD peptide with fluorescent dye and linker molecules (named SBD_f_ group) and Aβ_1–25_ SBD peptide without dye and linker (named SBD_p_ group). In each group, two variants of Aβ_1–25_ SBD were examined: wild type Aβ_1–25_ peptide (named K16) and K16E mutant Aβ_1–25_ peptide (named E16).

Several series of simulations were performed to find stable configurations of lipid-bound SBDs. Details of simulation setups are described in Experimental Section 4.1 and Supplemental Materials. Quantitative analysis results on SBD_f_ and SBD_p_ binding modes with GT1b gangliosides, such as salt bridges, CH–π interactions, and binding free energies, will be discussed in the following sections. All the data analysis was based on the fourth and fifth simulation series, *i.e.* with no distance constraints.

### 2.1. E16/K16 Sphingolipid Binding Domain (SBD) Variants Adopt Different Conformations

**Figure 2:**
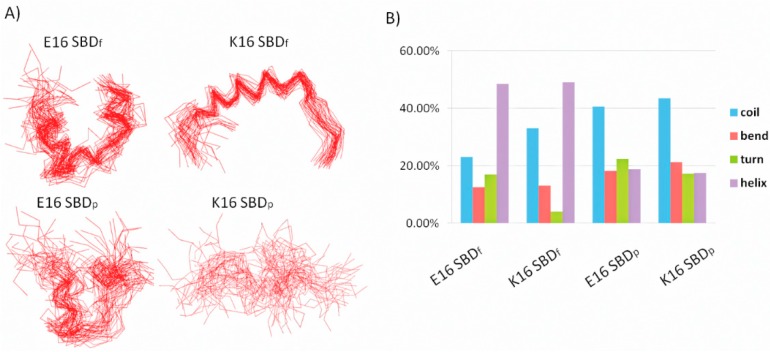
(**A**) Superimpositions of SBD configuration binding with GT1b during the simulations, one frame per 2 ns; (**B**) Secondary structure distributions of SBDs in the binding modes with GT1b. E16 SBD_f_; K16 SBD_f_; E16 SBD_p_; K16 SBD_p_

The simulations of E16 and K16 SBD_f_ suggested different conformations for the two variants, despite their identical initial configurations (Figure 2A). The configurations of E16 variants are helix-turn-helix loops, similar to the V3-like loops in Fantini’s model [19,52]. K16 variants, surprisingly, were in coil-helix-coil configurations and had larger contact area with ganglioside sugar groups than E16 variants.

With the fluorescence label, the conformational ensemble of SBD_f_ had much smaller structural fluctuations than those SBD_p_ as illustrated in Figure 2A. The proportion of unstructured coil-adopting residues decreased from ~40% to ~25% with dye and linker molecules. Besides, the proportion of helix-adopting residues doubled from ~20% to ~50% with the addition of the labels (Figure 2B). Root mean square fluctuation (RMSF) of SBDs binding with GT1b are shown in Figure 2C. Residues 7–16 show the structural stability in both SBD_f_ and SBD_p_ variants. Terminal regions of SBD_p_ are more flexible than SBD_f_. These results are in line with the output of our replica exchange MD simulations of SBD in the aqueous environment [20], suggesting that fluorescent dye and linker molecules enhance SBD folding and helical propensity.

### 2.2. SBD_p_ Peptides Are Capable of Binding to GT1b Clusters in Distinct Modes

**Figure 3:**
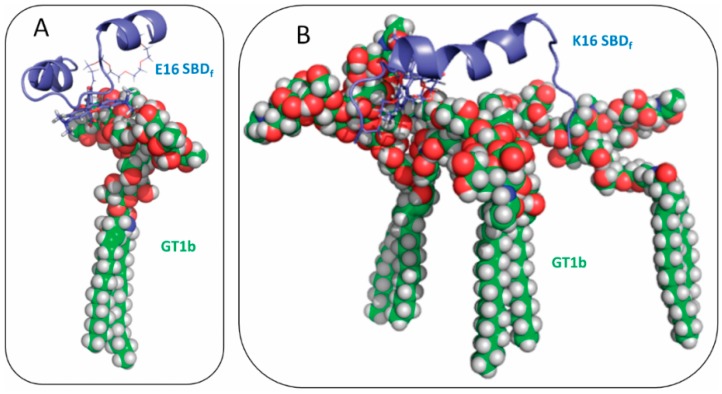
Binding modes of SBD_f_ with GT1b: (**A**) E16 variant; and (**B**) E16 variant. GT1b molecules are shown as green balls and the SBD molecule is represented by blue sticks.

According to our simulation results, the binding mode of E16 SBD with GT1b was as V3-loop configuration, which was in line with the model discovered by Fantini’s group [12,16]. As illustrated in Figure 3A, both the N-terminal and C-terminal residues of E16 SBD, E3-S8 and V18-D23, form the helically structured region. Residues Y10–H14 are in the turn region (also as suggested by Fantini *et al.* [21] ). It can be seen that the side chains of residue Y10 and H13 in this turn region participate in the interactions with GT1b sugar head group.

In addition, we found that K16 SBD_f_ peptides can interact with multiple GT1b molecules when the coil-helix-coil configuration is adopted (Figure 3B). Multiple GT1b molecules form clusters consisting of two or three members. The clusters provide receptor-like pockets or surfaces for binding with K16 SBD_f_. The middle residues R5-F19 of SBD formed a long helix, which bound with this platform of sugar rings from multiple GT1b molecules. In particular, residues such as R5, H13 and K16 form contacts with the sugar group cluster.

**Figure 4:**
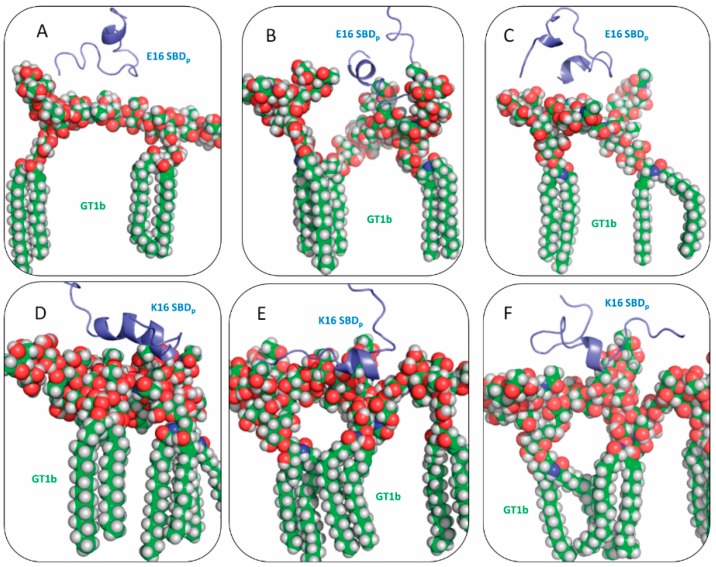
Three most frequent binding modes of SBD_p_ with GT1b: (**A**–**C**) E16 variant and (**D**–**F**) K16 variant.

Similarly to E16/K16 SBD_f_ peptides, individual SBD_p_ peptide molecules (without the dye and linker) were able to interact with clusters of GT1b. E16 SBD_p_ remained in the helix-turn-helix configuration. In Figure 4A–C, the three most populated E16 SBD_p_ binding modes are shown with probabilities of 37.9%, 17.6% and 11.5%, respectively. In these configurations, the number of helical residues decreased, but the C-terminal helical region from F19 to E22 remained intact. Residues R5, Y10 and H13 bound to GT1b sugar rings in a similar way as SBD_f_. For K16 SBD_p_, 59% of the population still adopted a coil-helix-coil configuration (Figure 4D), similar to K16 SBD_f_, and less helical conformation was observed. This was also true for the other binding modes shown in Figure 4E,F, corresponding to a probability of 7.1% and 7%, respectively. In these K16 SBD_p_ binding modes, the Y10-H13 region remained helical. Residues R5, H13 and K16 formed close contacts with multiple GT1b molecules.

We also examined SBD_f_ probes folding conformations in water. Both E16 and K16 showed the V-turned conformation (Figure S2). It suggests the mutation from Lys to Glu at residue 16 does not influence the SBD_f_ conformation significantly in water, but does in the folding patterns with GT1b ganglioside.

### 2.3. Residues R5, H13 and K16 Interact Electrostatically with Ganglioside Sialic Acid Groups

**Figure 5:**
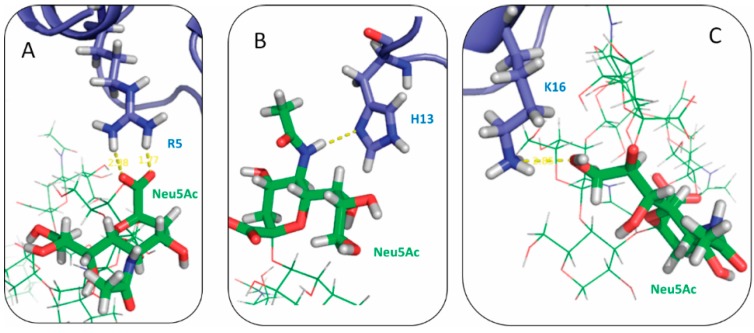
Snapshots of electrostatic interactions between positively charged residues of SBD in (**A**) arginine; (**B**) histidine; and (**C**) lysine, to Neu5Ac of GT1b gangliosides.

**Figure 6:**
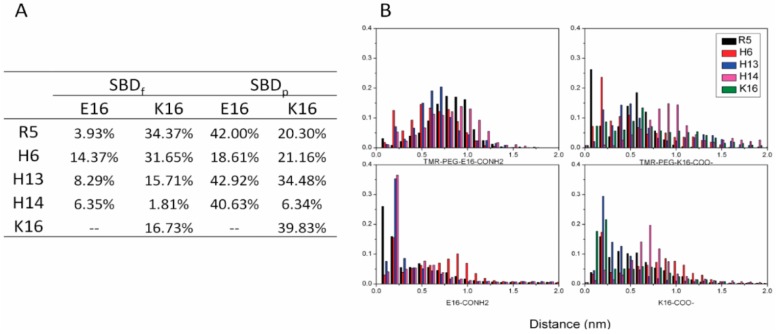
Salt bridge distances between SBD and GT1b during the last 100 ns of simulation: (**A**) probabilities of strong salt bridges (<0.35 nm); and (**B**) distance distributions of salt bridges.

At neutral pH, SBD contains five positively charged residues R5 H6, H13, H14 and K16. Three negatively charged Neu5Ac residues are included in GT1b sugar groups. The electrostatic forces between Neu5Ac and the positively charged amino acids were expected to be involved in the interactions between SBD and GT1b (Figure 5). Distributions of the distances between SBD positively charged residues and GT1b Neu5Acs were plotted for four binding modes (Figure 6).

Residue Arg5 was proposed to be important in the interaction with ganglioside sialic acid groups in Fantini’s model. In our simulation data, Arg5 was found to have a high probability to form salt bridges in K16 SBD_f_ and E16 SBD_p_ groups. Residue His13 was also critical in the binding of all variants except in E16 SBD_f_. Similarly, residue K16 was crucial to forming the salt bridge in both SBD_f_ and SBD_p_ groups.

### 2.4. Residue Y10 and F4 Formed CH–π Interactions with GT1b Sugar Groups in E16 and K16 SBD Variants Respectively

**Figure 7:**
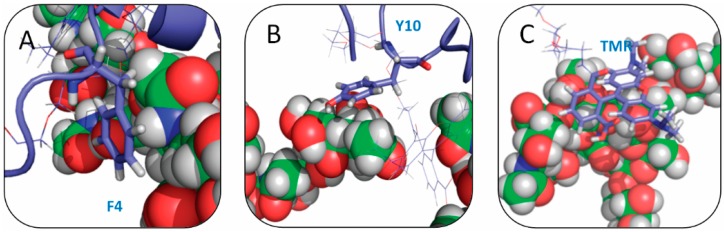
Snapshots of CH–π interactions between SBD at (**A**) phenylalanine 4; (**B**) tyrosine 10; and (**C**) TMR (tetra methyl rhodamine) and GT1b gangliosides.

**Figure 8:**
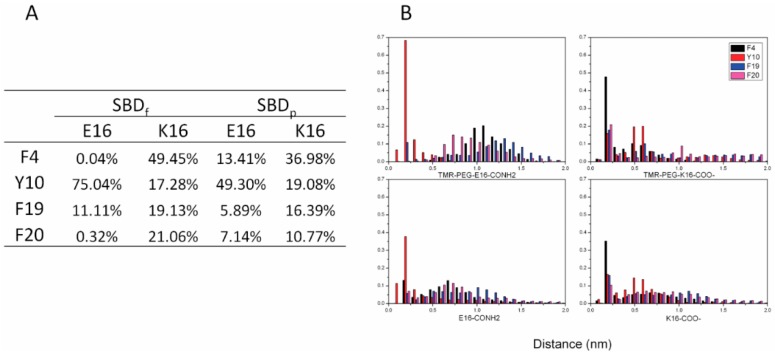
Distances of CH–π interactions between SBD and GT1b during last the 100 ns of simulation: (**A**) probabilities of CH–π interactions (<0.35 nm); and (**B**) distance distributions of CH–π interactions.

The aromatic side chains of SBD form CH–π interactions with the sugar rings of GT1b carbohydrate head groups, constituting the important mechanism of SBD-membrane binding. In SBD, there are four aromatic residues, F4, Y10, F19 and F20. In our simulations, we indeed found close contacts between aromatic side chains and sugar rings (Figure 7). Distance distributions between aromatic residues and sugar groups in the four binding modes were calculated to check for CH–π interactions (Figure 8). Clearly, residue Y10 of the E16 variants and F4 of the K16 variants form close contacts with GT1b sugar groups through CH–π interactions.

The different CH–π interaction patterns in E16 and K16 variants were consistent with the difference in their binding configurations. Residue Y10 was in the turn region of E16 helix-turn-helix loop, and CH–π interactions in this position were critical for the peptide configurations that interacted with GT1b.

### 2.5. Binding Energy Calculations Showed Residues R5, Y10, H13 in E16 Variants and Residues R5, H13, K16 in K16 Variants Played Important Roles in Binding Modes to GT1b

**Figure 9:**
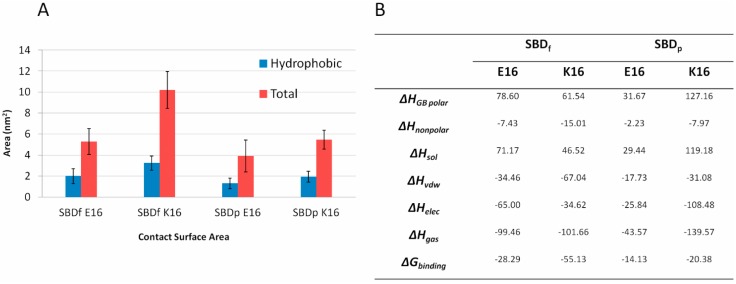
(**A**) Contact surface areas between SBD and GT1b; (**B**) Average MM-GBSA binding energies between SBD and GT1b (Kcal/mol).

The binding energies of four binding modes between SBD variants and GT1b gangliosides were calculated, employing the Molecular Mechanics/Generalized Born Surface Area (MM-GBSA) method. As shown in Figure 9, the binding energies were highly correlated with the contact surface area between SBD and GT1b. SBD_f_ had a lower binding energy with GT1b than SBD_p_ indicating the dye and the linker group contributed to GT1b binding.

**Figure 10:**
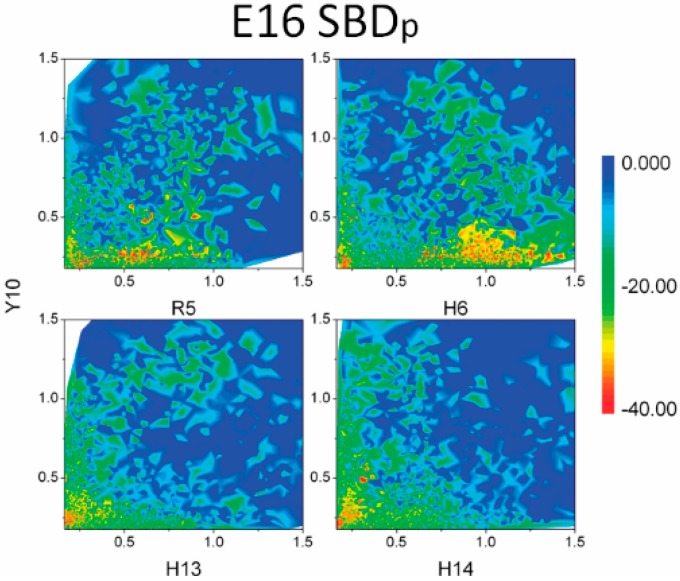
MM-GBSA binding energies (Kcal/mol) between SBD and GT1b gangliosides. Distances of salt bridge and CH–π interaction were set as two coefficients of variation. Distances of salt bridges between positive residues (R5, H6, H13 and H14) and Neu5Ac were set as the horizontal axis, and distances of CH–π interactions between aromatic rings of Y10 and CH groups in sugar rings were set as the vertical axis.

To reveal a clearer relationship between binding energies and electrostatic or CH–π interactions, a three dimensional map of binding energy was constructed as a function of two variables, distances of electrostatic and CH–π interactions (Figure 10 and Figures S3 and S4).

The binding energies were correlated with the formation of electrostatic and CH–π interactions. In SBD_p_ E16 variant as shown in Figure 10, most of the favored binding configurations (red and green, binding energy less than −20 Kcal/mol) were located within the region where the CH–π distance between Y10 and sugar groups was less than 0.4 nm. Regions favoring salt bridge formation involving R5, H13 and H14 residues also had relatively low binding energies. Similarly, in the case of SBD_p_ K16 SBD (refer to Figure S3) CH–π interactions from residues H13 and K16 showed correlation with binding energies, *i.e.*, regions having short CH–π distances were usually filled by configurations with low binding energies (red color). The CH–π interactions of both F4 and Y10 on the other hand, did not show good correlations with low binding energies, indicating the interaction between F4, Y10 and the sugar groups may not play a critical role in ganglioside binding. Similar effects on binding free energy were observed in SBD_f_ E16 and K16 variants (refer to Figure S4).

## 3. Discussion

Ganglioside- and sphingolipid-binding peptides were recently studied by several research groups [21,25,53,54]. Through surface pressure measurements and mutagenesis tools, Fantini and Yahi investigated the glycosphingolipid-binding motif of α-synuclein (KEGVLY_39_ VGSKTK) to different glycosphingolipids [25], where Y39 was found to be critical for glycosphingolipid binding. In addition, Aβ_5–16_ peptide, RHDSGY_10_ EVHHQK, was proposed to interact in a similar manner with glycosphingolipids [25]. Surprisingly, it was found that Y10A substitution in Aβ peptide (equivalent to position Y39A in α-synuclein) had no significant impact on glycosphingolipid binding. However, a double R5A, Y10A mutant SBD diminished binding to cells and membranes [20,38]. In the present study, the explanations were provided about the molecular mechanisms of the insensitivity in Y10A Aβ binding to glycosphingolipids. Fantini and Yahi performed molecular modeling studies on the recognition patterns between glycosphingolipids and α-synuclein [25]. E16 Aβ variants interacting with GT1b in our simulations is similar as the binding configuration of α-synuclein (shown in Figure 3A and Figure 4A–C). Such a binding mechanism can be described as Y-anchored requiring a helix-turn-helix motif; this is also applicable to HIV-1 surface envelope glycoprotein gp120 [22], human prion protein [23], as well as bacterial adhesins [55]. On the other hand, we found that the wild type Aβ_1–25_ peptides (the K16 species) do not employ the same binding motif with gangliosides. Instead, a coil-helix-coil motif was found to associate with the clusters of GT1b molecules (shown in Figure 3B and Figure 4D–F), In this binding mode, the middle part of the Aβ_1–25_ (usually in the helical conformation) forms extensive contacts with the GT1b clusters, and the residue Y10 is not located near the interface.

Thus, the seemly contradictory experimental data of the glycosphingolipid binding effects of Y39 mutation on α-synuclein and Y10 mutation on Aβ binding from Fantini and Yahi [18] can be rationalized based on the two binding modes we suggest here. The binding of α-synuclein to glycosphingolipids probably takes the Y39-anchored mode, unlike the Y10-anchored mode in the E16 Aβ variant. Y39 plays an important role in glycosphingolipid binding. On the other hand, the wild type Aβ interacts with glycosphingolipids via the coil-helix-coil motif, in which the Y10 residue has no critical contributions to the binding.

Interestingly, the K16E mutation affects the folding process of the peptide in simulations. According to our data, before tight binding to GT1b occurs, peptides have already finished the folding process, at around 100 ns (shown in Figure S5). Different folded structures, *i.e.*, helix-turn-helix for E16 variants and coil-helix-coil for K16 wild type, were formed and thereafter developed extensive interactions with GT1b molecules.

In addition, we found that SBD monomers usually bound with multiple GT1b molecules. Ganglioside clusters provide variant pockets/surfaces for binding. Without dye and linker molecules, SBD peptides with more random coil of secondary structure fit into the surface of ganglioside oligomers by making close contacts with multiple GT1b molecules through basic residues R5, H13 and K16. Such behavior is in line with the experimental observation that full-length Aβ specifically recognizes and binds to ganglioside clusters, which are thought to occur in membrane regions containing cholesterol and other sphingolipids [28,34], although the composition of its putative target membrane domains has not been characterized.

In Aβ_1–25_, at least one of two histidines at positions H13 and H14 were demonstrated to be involved in binding to the sialic acids of GM1 by NMR [56]. Residues 14–17 of Aβ_1–25_ were suggested to mediate binding in the report. Double mutagenesis on residues R5 and Y10 were demonstrated by model lipids and live cell studies both to severely impair binding [20,38]. Our simulation data also strongly support that the salt bridges in these residues play crucial roles in the SBD-GT1b membrane binding. Binding energy calculations suggest that residues R5 and H13 form salt bridges with Neu5Ac in both E16 and K16 variants.

The fluorescent dye and linker do not significantly change the binding configurations of SBD probes with gangliosides. Indeed, the dye and linker molecules enhance SBD_f_ peptide folding and stabilize the binding with GT1b. We also applied the simulations of SBD_f_ in aqueous solution. The cluster analysis of the structures of SBD_f_ E16 and K16 based on pairwise RMSD are shown in Figure S6. The SBD_f_ probes in water, both E16 (Figure S6A) and K16 (Figure S6B), show the V-turned conformation. This folding conformation remains in SBD_f_ E16 binding with GT1b, but changes to the coil-helical-coil conformation in K16 binding case, indicating the membrane environment exerts different influences depending on the peptide species.

In a neutral pH environment, SBD_f_ probe was designed as two variants of −4 or −5 charged in experimental work to demonstrate the charge effects of lipid raft probes [20,37,38]. GT1b is −3 charged in neutral pH environment. The electrostatic repulsion between them is the barrier for the spontaneous binding in short-time MD simulations. Hoshino *et al.* [51] performed the MD simulations of Aβ peptide binding with GM1-contained lipid raft domain. Long simulation time was needed for Aβ-GM1 spontaneous binding even though GM1 is −3 charged, and only 40% of monomer Aβ binding to GM1 were successfully observed within hundreds of nanosecond. Considering the difficulties of obtaining stable binding, we applied the *z*-axis position restraint to speed up SBD binding configurations searches. Ultimately, we also obtained 40% successful binding trajectories between SBD_f_ and the GT1b lipid raft domain.

## 4. Experimental Section

### 4.1. Modeling of the SBD Fluorescent-Tagged Probe and a GT1b Containing Plasma-Membrane-Like Bilayer

SBD peptide consisting of the sequence DAEFR_5_ HDSGY_10_ EVHHQ_15_ KLVFF_20_ AEDVG_25_ (derived from Aβ_1–25_) with tetra methyl rhodamine (TMR) fluorescent tag is referred to as SBD_f_. Linker molecules consisting of two copies of an amino-polyethyleneglycol (amino-ethoxy-ethoxy-acetyl; AEEAc2) or 4 copies of polyethylyneglycol (PEG4) were included in the sequence in the simulations, in order to duplicate what has been published in experimental data [20]. The TMR tag, previously used in FCS and SPR experiments to label the peptide [20], was included in the models as the fluorescent dye. The structural models of AEEAc2, PEG4 and TMR are shown in Figure S5. The partial charges of AEEAc2, PEG4 and TMR were calculated by Gaussian09 [57], using R.E.D.-III.4 tool [58]. Atom types were assigned by the antechamber program, which is included in AMBER10 package [59].

A plasma membrane-mimicking lipid bilayer system was built using 60 1-palmitoyl-2-oleoylphosphatidylcholine (POPC) molecules, 40 cholesterol (CHOL) molecules, and 16 sphingomyelin (18:0) (SM) molecules, of which the molar concentration was around POPC:CHOL:SM=15:10:4, in keeping with reported values for typical cellular plasma membranes [60]. Additionally, 4 different percentages of GT1b, corresponding to molar concentrations of 0%, 4%, 8%, or 12% were added to the bilayers. The lipid systems were solvated in a water box containing ~7000 water molecules. SBD_f_ probe was added to the lipid bilayer systems containing different concentrations of GT1b. Random initial position and orientation of SBD_f_ was applied. The initial distance between outer leaflet of the bilayer and the probe was set to 1 nm. Different numbers of Na^+^ ions were added to neutralize the system. Each system was equilibrated for 50 ns. Numbers of Na^+^ ions in 0%, 4%, 8%, or 12% GT1b lipid bilayer with E16 SBD_f_ probe was 6, 18, 30, or 42, respectively. And Numbers of Na^+^ ions in 0%, 4%, 8%, or 12% GT1b lipid bilayer with K16 SBD_f_ probe was 4, 16, 28, or 40, respectively.

Several series of simulations were performed to find stable configurations of lipid-bound SBDs. Detailed information was described in Supplemental experimental procedures. Briefly, in the first series simulations, different concentrations of GT1b molecules (12%, 8%, and 4%) in lipid membrane were tested. Eventually 4% was chosen as the GT1b concentration in the following simulations. In the second series, single SBD_f_ was added above 4% GT1b raft-like lipid bilayer and simulated for 200 ns, no stable binding event between SBD_f_ and GT1b was observed. Thus in the third series, C-terminus of SBD_f_ was restrained at the surface of membrane and simulated for 200 ns, which means SBD_f_ can only slide on the membrane surface. SBD_f_ formed stable interactions with GT1b (Figure S6). Next, in the fourth series, restraining of C-terminus of SBD_f_ was removed and SBD_f_ could freely move outside the membrane. The simulations were lasted for 200 ns and in two trajectories each of E16 and K16 variants the binding between SBD_f_ and GT1b were remained eventually. In the fifth series, fluorescent dye and linker molecules were removed, therefore the SBD_p_ were simulated with 4% GT1b raft-like lipid bilayer for 200 ns.

All the simulations were run using the Gromacs 4.5 program [61,62,63]. Amber 99SB force-field parameters [64] were used in this study, and a GLYCAM06 force-field [65] was used for sugar head groups of gangliosides. The SPC/E (extended simple point charge) model [66] was used for water molecules. The secondary structure of the protein was determined by the DSSP program [67]. Lipid parameters were optimized in AMBERTOOLS and the atom charges were calculated by RESP charge [68].

A periodic boundary condition (PBC) was applied to the system to minimize the boundary effects. The V-rescale coupling [69] was used to maintain the temperature at 325 K, above the transition temperature of the lipid mixture in the bilayer. Semi-isotropic Parrinello–Rahman pressure coupling [70] with a pressure of 1.0 atm was used in this study. The pair-list of non-bonded interactions was recalculated every 10 time-steps with a pair-list cut-off distance of 10 Å. The particle mesh Ewald (PME) method [71] was used for full evaluation of long-range electrostatic interactions. The LINCS routine [72] with a tolerance of 0.0004 was used to constrain all bond lengths with hydrogen atoms in all simulations. The atomic coordinates were saved every 1 ps for subsequent data analysis.

### 4.2. Measurement of Electrostatic and CH–π Interactions

The electrostatic interactions between positive charge residues of SBD and negative charged NeuAc5 groups of GT1b gangliosides were monitored by measuring the minimum distance between atoms on the side chain of the amino acids and atoms on the Neu5Ac ring of GT1b molecules. CH–π interactions between aromatic residues in SBD and GT1b were checked by measuring the minimum distance between the atoms on the side chain ring of the amino acid and the atoms on the carbohydrate ring of the neighboring GT1b molecules. Both the criteria adopted for counting salt bridges and CH–π interactions were that the distances between function groups were smaller than 0.35 nm.

### 4.3. MM-GBSA Binding Energy Calculations

In a MM-GBSA scheme, binding free energy *∆G_bind_* is given by

(1) $$∆G_{bind}=∆G_{gas}+∆G_{sol}-T∆S$$

where Δ*G_gas_* is the gas phase enthalpic contribution, Δ*G_sol_* is the desolvation free energy upon binding, and *-T*Δ*S* is the entropic distribution. Δ*G_gas_* is the energy difference between the binding complex and the separated receptor and ligand molecules, consisting of van der Waals (Δ*G_vdw_*) and electrostatic interactions (Δ*G_ele_*). Δ*G_sol_* is the solvation free energy difference between the bound complex and the two separated molecules. It can be divided into the electrostatic (Δ*G_GBpolar_*) and non-polar (Δ*G_GBnonpolar_*) contributions. The electrostatic term is calculated by a Generalized Born (GB) model [73] with the implicit water treatment. The non-polar term is calculated from the solvent accessible surface area. The entropy contribution, *-T*Δ*S*, was not considered since the entropy calculations in MM-GBSA were reported to have large deviations and give unreliable results [74,75]. MM-GBSA binding energies were calculated as the average values of every 100 frames to reduce the energy fluctuation.

## 5. Conclusions

In this study, we simulated Aβ_1–25_ SBD monomers interacting with GT1b containing raft-like lipid bilayers with fluorescent dye and linker molecules. SBD peptides with and without a fluorescent tag are capable of binding with GT1b gangliosides via electrostatic and CH–π interactions. Two binding modes are found, one being the Y10-anchored binding mode, which is related to the well-known helix-turn-helix motif; the second, a new binding mode, involves a loop-helix-loop motif and is found to best describe wild type Aβ_1–25_ binding to clustered GT1b gangliosides. The fluorescent dye and linker molecules, which were part of the SBD probe construction, do not change the SBD binding motif to gangliosides. Understanding the details of SBD interacting with lipid rafts could be beneficial for the development of therapeutic interventions in Aβ’s pathogenic effects. For example, such knowledge could help to develop inhibitors of Aβ’s interaction with gangliosides and raft domains in membranes, which could reduce its toxic effects on neurons.

## Supplementary Materials

Supplementary materials can be found at http://www.mdpi.com/1422-0067/16/11/25955/s1.
