# Supplementary material for: Aβ1-25-Derived Sphingolipid-Domain Tracer Peptide SBD Interacts with Membrane Ganglioside Clusters via a Coil-Helix-Coil Motif
Source: Int J Mol Sci. 2015 Nov 3;16(11):26318–32. doi: 10.3390/ijms161125955 (PMC4661814; doi:10.3390/ijms161125955)
Supplement: Supplementary file 1 [file ijms-16-25955-s001.pdf]

International Journal of
Molecular Sciences

MDPI

Supplemental Information

1. Simulation Results of GT1b Containing POPC Bilayers

Area per lipid, bilayer thickness, and lateral diffusion rate were used to analyze the quality of raft-like lipid bilayer model, as shown in Table S1. Overall vertical distances between phosphate groups on both lipid sides were calculated as the thickness of the lipid bilayer.

Area per lipid for the raft-like lipid bilayer systems were ~0.38 nm^2, lower than that of the pure POPC lipid bilayer (~0.7 nm^2), agreeing with the more compact state of the raft membranes. Consistent with this, the bilayer thicknesses of the raft-like membranes were ~5.5 nm, compared to the pure POPC bilayer at 3.68 nm. Finally, the lateral diffusion rates of raft-like lipid bilayers (from 0.053 to 0.119 $\mu m^2/s$) were, as expected, lower than those of pure POPC (0.45 $\mu m^2/s$), in agreement with a more ordered and viscous state [76].

Compared to the raft-like POPC bilayer with 5% GalCer constructed by Hall *et al.* [77], our model with 12% GT1b group was similar in area per lipid (0.39 nm^2 *vs.* 0.41 nm^2) and lateral diffusion rate (0.053 $\mu m^2/s$ *vs.* 0.047 $\mu m^2/s$). Our bilayer thickness was larger (5.33 nm *vs.* 4.56 nm), likely owing to the larger head group of GT1b vs. GalCer.

2. Low GT1b Concentrations Bound Better to Sphingolipid Binding Domain (SBD) Probe than Higher Concentrations

SBD peptide TMR-AEEAc-E16-COO- was added to lipid bilayers with different concentrations of GT1b (12%, 8%, 4% and 0%). The SBD remained far away from the bilayer in all systems. Throughout four trials of 20 ns simulations, the minimum distance between SBD and the bilayer remained over 1.0 nm except for one trajectory on 4% GT1b and two trajectories on 0% GT1b, as shown in Figure S1. Our simulations showed that only with low GT1b concentrations there exists an attraction between SBD and the membrane, and SBD makes close contact with the lipid bilayer. One trajectory on the 4% GT1b system showed SBD binding onto the bilayer. At GT1b molar concentration of 4% and above, the minimum distances between SBD and lipids seen in trajectories were seldom below 0.5 nm, indicating a repulsive interaction between SBD and negatively charged gangliosides in the lipid bilayer.

In the following study, the lipid bilayer with 4% GT1b was chosen as a representative bilayer to test interactions with different SBD variants.

3. Positive Charges Enhanced Helical Content of SBD Probe on the Lipid–Water Interface

Four different SBD_f models/groups were built, as listed below:

(a) SBD_f 1: TMR-AEEAc2-SBD-E16-COO-
(b) SBD_f 2: TMR-PEG4-SBD-E16-COO-
(c) SBD_f 3: TMR-PEG4-SBD-E16-CONH2
(d) SBD_f 4: TMR-PEG4-SBD-K16-COO-

Several series of simulations were performed to find stable configurations of lipid-bound SBDs.

In the first series, each SBD_f molecule was placed 1.0 nm away from the lipid bilayer as an extended structure. Fifty nanosecond simulations were performed for each of the four GT1b concentrations. In total, 800 ns (4 × 4 × 50 ns) trajectories were collected and the distribution of minimum distance between SBD_f and lipids are shown in Figure S1. Unexpectedly, binding events (defined by a minimum distance between SBD and lipids being less than 0.5 nm) were only found in the systems with moderate amounts of GT1b, at 0% and 4%. In systems with 8% or 12% GT1b, SBD_f molecules did not approach the lipid bilayer closer than 1 nm, indicating there is net repulsion between SBD and the lipid bilayer. Thus, the lipid system with 4% GT1b was chosen for the following study.

In the second series of MD simulations, data for each SBD_f interacting with 4% GT1b lipid bilayer were collected for 200 ns. Initially each SBD_f molecule was placed at a distance 1.0 nm away from the lipid bilayer in an extended structure. Five trajectories for each of the four SBD_f variants were launched, resulting in a total of 4000 ns (4 × 5 × 200 ns) of simulation data. The SBD_f variants were found to be attracted to GT1b head groups for a short time (less than 300 ps) after which they were repelled from the membrane. This observation indicated that the binding affinity between SBD and GT1b was not strong (at physiological pH = 7). Stable binding modes for SBD_f with lipids was expected to require much longer simulation times due to the repulsion.

Given the difficulty of running full atomistic molecular dynamics simulations beyond the hundred nano-second time scale, direct simulations where actual binding between SBD_f in aqueous solution onto a lipid bilayer may not be feasible. In order to speed up the binding conformation searching, we attempted to limit the motion of SBD_f, from three dimensions to two, by letting it only slide on the lipid surface.

The third series of simulations also began with each of the four SBD_f molecules being placed at a distance 1.0 nm away from the lipid bilayer in an extended structure. However, a restraining force on the Z-direction was applied onto the C-terminal of the SBD_f, residue G25. Under this restraint, the residue G25 was constrained in the Z-direction to within 0.2 nm above the XY plane where the phosphate headgroups of lipids lay. Five trajectories with different initial velocities for each system were performed for 200 ns, resulting in a total of 4000 ns (5 × 4 × 200 ns) data. The SBD_f C-terminal here could not travel away from the phosphate XY-plain, but could slide freely on the bilayer surface. After 200 ns constrained simulations, all four SBD_f variants bound to ganglioside sugar groups with a minimum distance between SBD_f and sugar groups of less than 0.5 nm, indicating interactions between SBD_f and GT1b. The putative binding models found in the restrained simulations up to this point underwent further checking through free MD simulations without the Z-direction constraints.

The fourth series of simulations were a continuation of the third series of simulations, however, the distance constraints were removed. Each trajectory ran for 200 ns resulting in a total of 4000 ns (5 × 4 × 200 ns) of trajectory. At the end of the simulations, SBD_f remained at the lipid-water interface, binding with GT1b sugar groups in only two of the five trajectories of TMR-PEG4-E16-NH2, and two of the five trajectories of TMR-PEG4-K16-COO-. These two groups of SBD_f have charge states of −5 and −4, respectively. Both are less negative than TMR-linker-E16-COO- groups (–6), which did not remain bound. Thus they have less repulsive electrostatic interaction with the negatively charged GT1b in the lipid bilayer. These two groups will be referred to as E16 SBD_f and K16 SBD_f.

The fifth series of simulations were the continuation of the four stable bound trajectories in the fourth series, with the dye TMR and the linker PEG4 removed to assess possible effects of the fluorescent dye TMR and the linker molecules on GT1b binding. We refer to these two groups of $A\beta_{1–25}$ peptides as SBD peptide without tags (SBD_P) E16-NH2 and K16-COO-. Each simulation ran for 200 ns.

Table S1. Parameters of lipid rafts during the simulation.

GT1b Concentration	Area per Lipid (nm^2)	Bilayer Thickness (nm)	Lateral Diffusion (μm^2/s)
0%	0.38 ± 0.01	5.43 ± 0.2	0.12 ± 0.03
4%	0.36 ± 0.01	5.76 ± 0.1	0.090 ± 0.02
8%	0.39 ± 0.02	5.36 ± 0.2	0.079 ± 0.02
12%	0.39 ± 0.03	5.33 ± 0.3	0.053 ± 0.02
5% GalCer &	0.41 ± 0.003	4.56 ± 0.02	0.047 ± 0.003
pure POPC bilayer *	0.71 ± 0.02	3.68 ± 0.1	0.45 ± 0.03

& simulation data from reference [1]; * data from our previous simulation. Errors are standard deviation of paralleled trajectories.

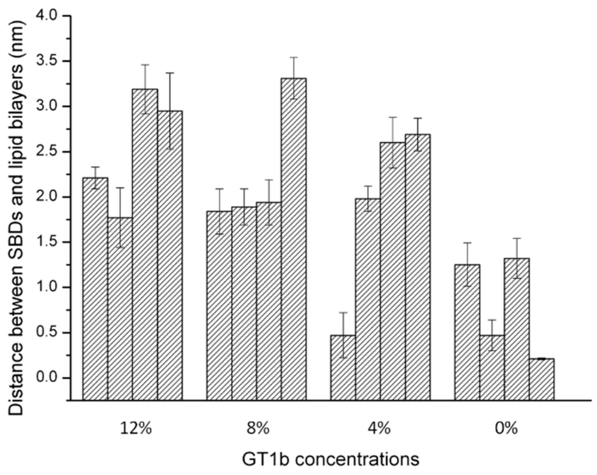

Figure S1. Minimum distances between sphingolipid binding domain (SBDs) and lipid bilayers: Four trajectories at each GT1b concentration were plotted, averaged over the last 1 ns. Errors are standard deviation of paralleled trajectories.

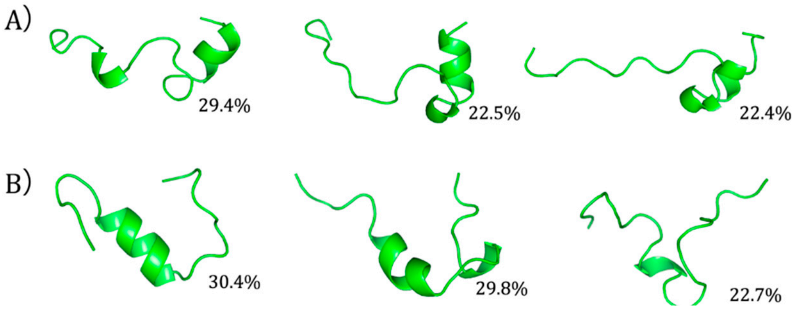

Figure S2. Top three conformations in structural cluster analysis of (**A**) SBD$_f$ E16; and (**B**) SBD$_f$ K16 in water solution. Fluorescent dye and linker molecules are not shown.

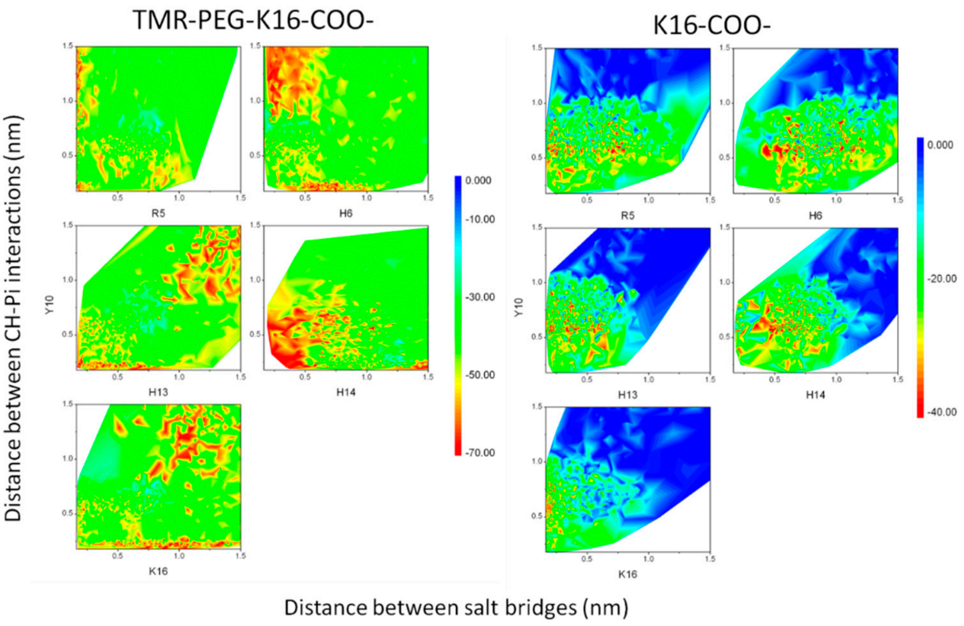

Figure S3. Binding free energy (kcal/mol) between K16 SBDs variants and GT1b gangliosides. Distances of salt bridges and Y10 CH–π interaction were set as two coefficients of variation. Distances of salt bridges between positive residues and Neu5Ac were set as the horizontal axis, and distances of CH–π interactions between aromatic rings of residues and CH groups in sugar rings were set as the vertical axis.

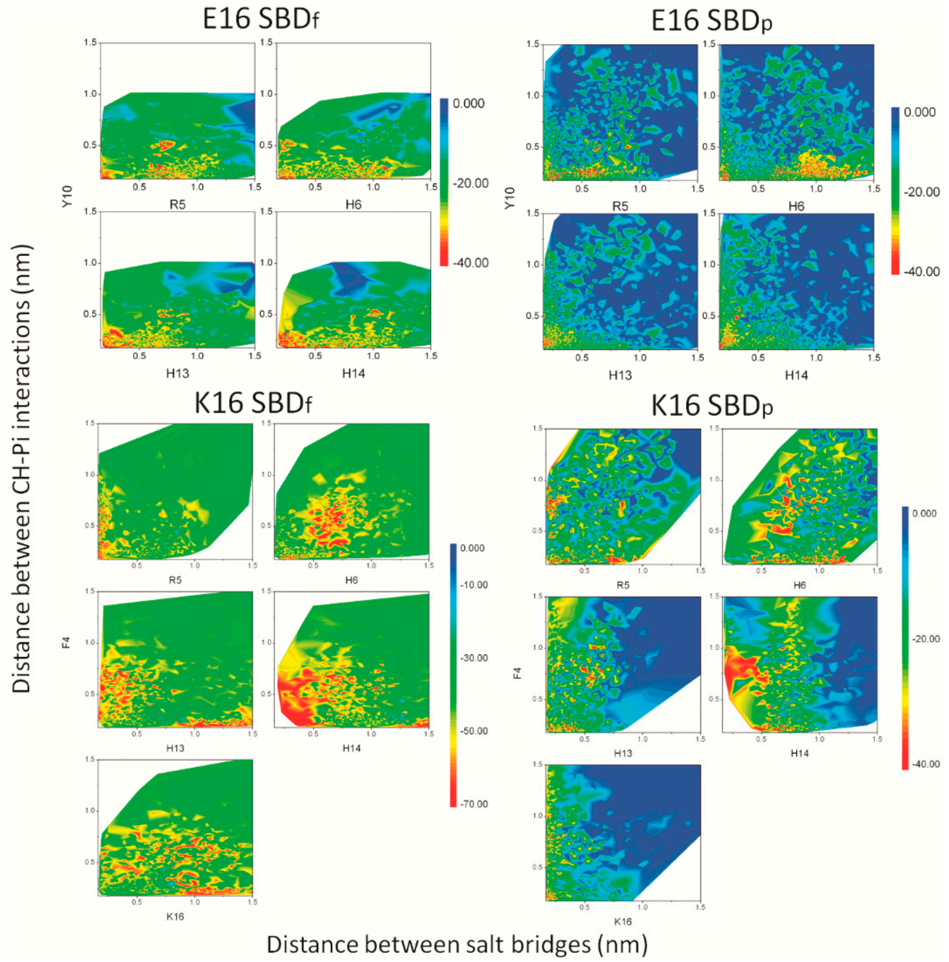

Figure S4. Molecular Mechanics/Generalized Born Surface Area (MM-GBSA) binding energies (kcal/mol) between SBD variants and GT1b gangliosides. Distances of salt bridge and CH–π interaction were set as two coefficients of variation. Distances of salt bridges between positive residues and Neu5Ac were set as the horizontal axis, and distances of CH–π interactions between aromatic rings of residues and CH groups in sugar rings were set as the vertical axis.

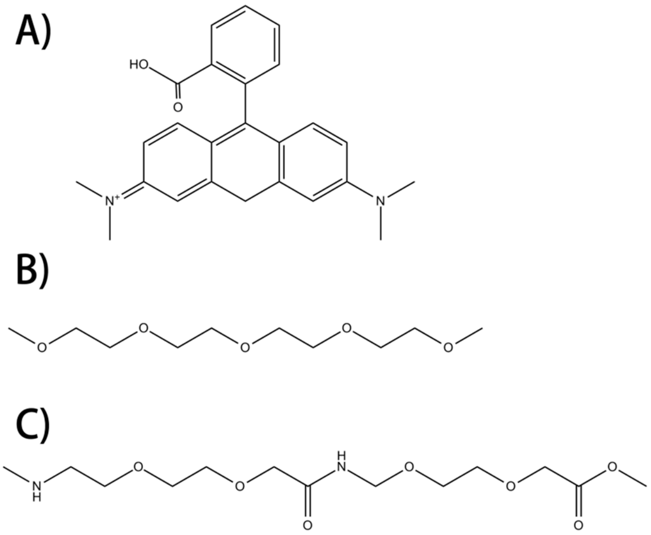

Figure S5. Chemical structures of (**A**) tetra methyl rhodamine (TMR); (**B**) four copies of polyethylyneglycol (PEG4); and (**C**) two copies of amino-ethoxy-ethoxy-acetyl (AEEAc2).

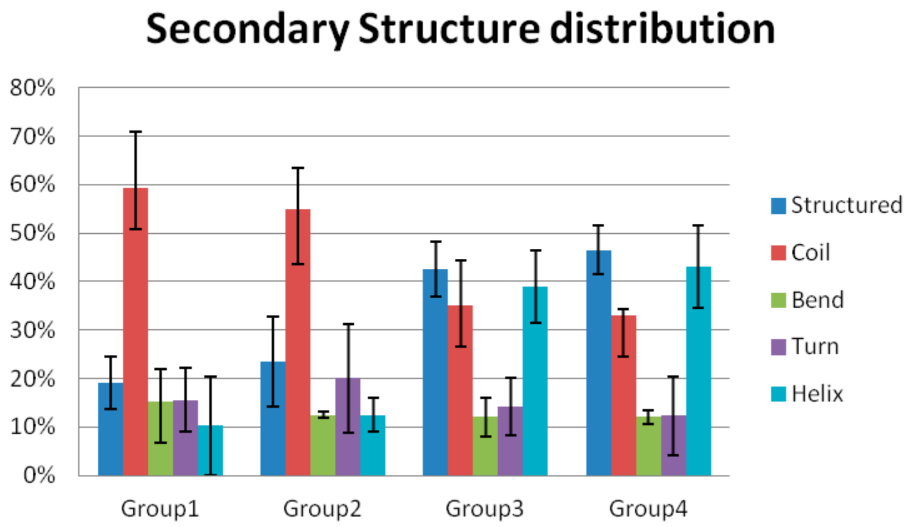

Figure S6. Secondary structure distributions of SBD_f in position constraint simulations (averaged over the last 10 ns). Errors are standard deviation of paralleled trajectories.
